# Anti-Inflammatory Effect of 3-*O*-[(6'-*O*-Palmitoyl)-β-d-glucopyranosyl Sitosterol] from *Agave angustifolia* on Ear Edema in Mice

**DOI:** 10.3390/molecules191015624

**Published:** 2014-09-29

**Authors:** Elizabeth Hernández-Valle, Maribel Herrera-Ruiz, Gabriela Rosas Salgado, Alejandro Zamilpa, Martha Lucia Arenas Ocampo, Antonio Jiménez Aparicio, Jaime Tortoriello, Enrique Jiménez-Ferrer

**Affiliations:** 1 Centro de Investigación Biomédica del Sur, Instituto Mexicano del Seguro Social (IMSS), Argentina No. 1, Xochitepec, Morelos CP 62790, Mexico; E-Mails: eli_valle_7@hotmail.com (E.H.-V.); cibis_herj@yahoo.com.mx (M.H.-R.); azamilpa_2000@yahoo.com.mx (A.Z.); jtortora2@yahoo.es (J.T.); 2 Centro de Desarrollo de Productos Bióticos, Instituto Politécnico Nacional (IPN), Km 6 carr. Yautepec-Jojutla, calle Ceprobi No. 6 col. San Isidro, Yautepec, Morelos CP 62731, Mexico; E-Mails: arenasocampoml@gmail.com (M.L.A.O.); aaparici@ipn.mx (A.J.A.); 3 Facultad de Medicina, Universidad Autónoma del Estado de Morelos, Leñeros s/n. Col. Volcanes, Cuernavaca, Morelos CP 62350, Mexico; E-Mail: gabyrosas62@hotmail.com

**Keywords:** *Agave angustifolia*, 3-*O*-[(6'-*O*-palmitoyl)-β-d-glucopyranosyl] sitosterol, anti-inflammatory

## Abstract

In Mexico *Agave angustifolia* has traditionally been used to treat inflammation. The aim of this study was to measure the anti-inflammatory effect of the extract of *A. angustifolia*, the isolation and identification of active compounds. From the acetone extract two active fractions were obtained, (AsF13 and AaF16). For the characterization of pharmacological activity, the acute inflammatory model of mouse ear edema induced with TPA was used. The tissue exposed to TPA and treatments were subjected to two analysis, cytokine quantification (IL-1β, IL-6, IL-10 and TNF-α) and histopathological evaluation. The active fraction (AaF16) consisted principally of 3-*O*-[(6'-*O*-palmitoyl)-β-d-glucopyranpsyl] sitosterol. In AaF13 fraction was identified β-sitosteryl glucoside (**2**) and stigmasterol (**3**). The three treatments tested showed a concentration-dependent anti-inflammatory effect (AaAc E_max_ = 33.10%, EC_50_ = 0.126 mg/ear; AaF13 E_max_ = 54.22%, EC_50_ = 0.0524 mg/ear; AaF16 E_max_ = 61.01%, EC_50_ = 0.050 mg/ear). The application of TPA caused a significant increase on level of IL-1β, IL-6 and TNFα compared with basal condition, which was countered by any of the experimental treatments. Moreover, the experimental treatments induced a significant increase in the levels of IL-4 and IL-10, compared to the level observed when stimulated with TPA. Therefore, the anti-inflammatory effect of *Agave angustifolia*, is associated with the presence of 3-*O*-[(6'-*O*-palmitoyl)-β-d-glucopyranosyl] sitosterol.

## 1. Introduction

The Mayans were probably the ethnic group that tamed the *Agave angustifolia* Haw, (Babki, chelem ki, henequén bab ki, chelem, chelemki henquén kij, “maguey espadín”, “maguey de Monte”) that belongs to the Agavaceae family. This specie together with the henequen (*Agave foucroydes* Lem.), have remained to date due to its economic and cultural importance in the Yucatan Peninsula (Southeastern Mexico). The traditional uses of agaves in Mexico have been for the production of fermented beverages, textile fibers, for construction and in medicinal applications [1,2]. Currently, the main use is in the production of alcoholic beverages (like tequila and mezcal), although the medicinal use has persisted in rural communities [2,3]. Different species of Agaves (*A. salamiana*, *A. mezcalensis*, *A. americana*) are used for treatment of illness associated with an inflammatory process as: stomach ulcers, wounds, gastritis, psoriasis and other rashes [4].

*A. angustifolia* is used for the elaboration of “bacanora” an alcoholic beverage from Sonora (North of Mexico) [5] and the other hand this specie is used in the “mescal region” in the South of Mexico (Oaxaca) for the production of the mezcal. Also, *A. angustifolia*, is used in the Mexican traditional medicine for the treatment of sprains or broken bones [6,7]. The chemical and pharmacological antecedents of this plant indicate that form the *A. angustifolia* leaves it has been isolated triterpene and steroidal saponins, either as aglycones or glycosylated compounds [8], such as, the steroidal saponin: 25*R*-5α-spironstan-12-one-3β-ol-3-*O*-[β-d-xylopyranosyl-(1→4)-β-d-galactopyranosyl-(1→3)-{β-d-xylopyranosyl-(1→3)-β-d-glucopyranosyl-(1→2)}-β-d-glucopyranoside], which was isolated from the methanol extract, and their saponin fraction provoked a marked anti-inflammatory, analgesic, schistosomicidal, cercaricidal and miracidicidal activities [8].

There is few data on the biological activity of *A. angustifolia*. In the present work we are reporting the modulator effect of the different chemical fractions as well as the active compound isolated from this plant 3-*O*-[(6'-*O*-palmitoyl)-β-d-glucopyranosyl] sitosterol (**1**), on the concentration of several cytokines (IL-1β, IL-4, IL-6, IL-10 and TNFα), by using the auricular edema induced by TPA model in mice.

## 2. Results and Discussion

### 2.1. Chemical Analysis

**Figure 1:**
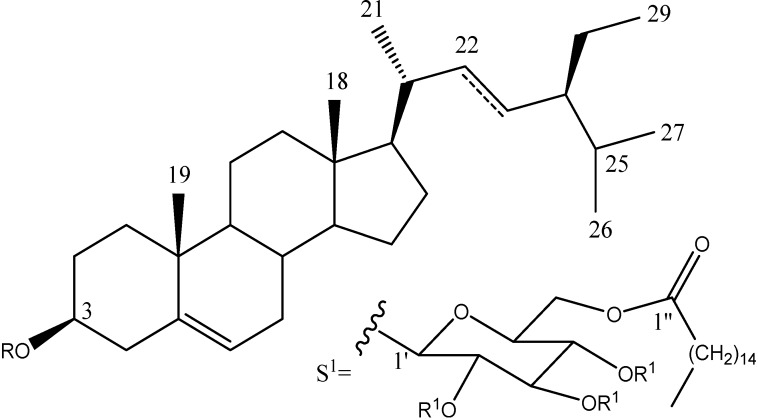
Bioactive chemical compound present in *Agave angustifolia*. At the bottom, the substituents which determine the identity of the compounds. (**1**) phytosteryl glucoside and its peracetylated derivative (**1a**), (**2**) β-sitosteryl glucoside and (**3**) stigmasterol.

Figure 1 shows the structures of the compounds present in the most active fraction (AaACF16) which was isolated chromatographically the phytosteryl glucoside **1**. The ^1^H- and ^13^C-NMR spectra of this peracetylated compound **1a** displayed signals for three units: a C29 steryl, a glucopyranose and a fatty acid. The EI mass spectrum of the per acetylated derivative **1a** showed a fragment due to a 3-deoxysitosterol (*m/z* 397) unit. The ^1^H-NMR spectrum exhibited signals for six methyls: two singlets at d 0.67 and 0.98; three doublets at 0.81 (*J* = 6.7 Hz), 0.83 (*J* = 7.2 Hz), 0.92 (*J* = 6.4 Hz), and one was a triplet at 0.85 (*J* = 7.2 Hz). The signal at 5.36 revealed the presence of a trisubstituted doble bond. This sterol structure was confirmed by the ^13^C-NMR data which were in agreement with those described in the literature [9]. COSY experiment allowed us to stablish the complete spin system of the monosaccharide: Anomeric proton was placed at 4.56 (d, *J* = 7.6 Hz, H-1') which is coupled with signal at 4.95 (dd, *J* = 8, 9.6 Hz, H-2'), H-3' was found at 5.20 (dd, *J* = 9.6, 9.6 Hz) which is coupled with signal at 5.04 (dd, *J* = 9.6, 9.6 Hz, H-4'). H-5' was found at 3.67 (m) couplet to the signals at 4.13 (dd, *J* = 2, 12 Hz) and 4.22 (dd, *J* = 5.6, 12 Hz) which correspond to the H-6 methylene group. These methylene signals were shifted by 0.6 ppm with regard to the values of a free therminal glucopyranose suggesting this position contains the esterificated fatty acid. Electronic impact mass spectrum of the hydrolyzed product displayed a molecular peak [M+] at *m/z* 240 which was assigned to the deoxy-C16 fatty acid part. This phytosterol is the 3-*O*-[(6'-*O*-palmitoyl)-β-d-glucopyranosyl] sitosterol. Although this compound has been previously described in other species, it is the first time that is was found in Agave specie.

**Table:** 

		R	R^1^
**1**	---	S^1^	H
**1a**	---	S^1^	Ac
**2**	---	Glc	---
**3**	∆^22^	H	---

### 2.2. Effect of A. angustifolia on 12-Ortho-Tetradecanoylphorbol-13-Acetate (TPA)-Induced Mouse Ear Edema Assay

**Figure 2:**
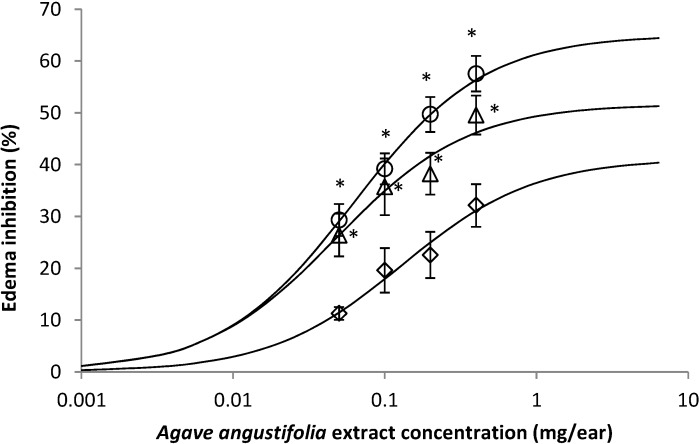
Concentration-response curve of extract AaAc (◇), AaF13 (mixture of β-sitosteryl glucoside and stigmasterol; (∆) and AaF16 (3-*O*-(6'-*O*-palmitoyl)-β-d-glucopyranosyl sitosterol; (◯) fractions from *Agave angustifolia* over edema inhibition (%), in mouse inflammation-TPA model. Dexamethasone had an anti-inflammatory effect above 80%, a result not shown. ANOVA *post hoc* Tukey with *n* = 6, * *p* < 0.05 when are compared with AaAc (◇) group.

The extract AaAc, induced an inhibition of inflammation by TPA in a concentration-dependent form (Figure 2, ◇), the major activity was obtained with the highest concentration used, 0.8 mg/ear. The mixture of compounds (β-sitosteryl glucoside (**2**) and stigmasterol (**3**)) in the AaF13 and the isolated compound AaF16 3-*O*-[(6'-*O*-palmitoyl)-β-d-glucopyranosyl] sitosterol, exhibited a likewise activity of the AaAc, however the inhibition percentage was higher for AaF13 (Figure 2, ∆) and even greater for AaF16 (Figure 2, ◯). Any data from the three treatments were statistically different with the group with DEX to 0.1 mg/ear (*p* > 0.05). Data from analysis of the concentration-response curve established that: AaAc extract present an E_max_ of 33.10% and EC_50_ of 0.126 mg/ear; AaF13 present an E_max_ 54.22% and EC_50_ of 0.0524 mg/ear and finally for AaF16 an E_max_ of 61.01% and EC_50_ was of 0.050 mg/ear.

### 2.3. Effect of A. angustifolia on the Concentration of Cytokines from Mouse Ear

The index level of each cytokine in the homogenates of mouse ears, are shown in the Table 1. The application of TPA caused a significant increase (*p* < 0.05) on the index level of IL-1β, IL-6 and TNFα, being 14.4, 7.3 and 12.4 times higher than the one of the basal group. The index levels for IL-4 and IL-10 were not significantly different from the basal control (*p* < 0.05). The control treatment with dexamethasone decreased the indexes of pro-inflammatory cytokines in 64%, 75% and 83% in the same order, these data was statistically different from the TPA group (*p* < 0.05), and for IL-4 and IL-10, there was no statistical differences (*p* > 0.05). Treatment with AaAc extract, AaF13 and AaF16 from *A. angustifolia* were able to provoke a decrement in the concentration rates of IL-1β, IL-6 and TNFα, such changes were dependents of the increase of the concentration.

Treatments from *A. angustifolia* (Table 1) showed a significant decrease on the index of IL-1β, IL-6 and TNFα, compared to negative control (TPA, *p* < 0.05). The dose of 0.8 mg/ear of AaF16 (3-*O*-(6'-*O*-palmitoyl)-β-d-glucopyranosyl sitosterol), induces a major effect than DEX by the concentration of IL-1β and TNFα, and the other hand the level index of IL-4 and IL-10, with the same treatment, are in the same range as in basal condition values (*p* > 0.05). But, in comparison with the negative control group (TPA), this dose of 3-*O*-(6'-*O*-palmitoyl)-β-d-glucopyranosyl sitosterol, induced a significant increase of IL-4 and IL-10 with 3.2 and 4 times, respectively, over the value obtained in the negative control (TPA, *p* < 0.05). In the same way, this increase was statistically significant when compared with the positive control group (dexamethasone, *p* < 0.05).

**Table 1:** Rate of increment of different cytokines concentration, in mouse ear with TPA.

Treatment	Concentration (mg/ear)	IL-1β	IL-4	IL-6	IL-10	TNF-α
**TPA**	0.00025	14.4 ± 0.9	0.9 ± 0.1	7.3 ± 0.6	0.7 ± 0.6	12.4 ± 0.6
**DEX**	0.1	5.1 ± 1.1 *	1.3 ± 0.2	1.8 ± 0.2 *	0.9 ± 0.3	2.1 ± 0.4 *
**AaAc**	0.05	10.4 ± 1.2 *	1.1 ± 0.1	7.1 ± 0.6 *	0.8 ± 0.1	10.9 ± 1.9
	0.10	9.7 ± 1.5 *	1.1 ± 0.1	6.9 ± 0.6 *	0.8 ± 0.1	10.3 ± 1.8
	0.20	9.4 ± 1.2 *	1.0 ± 0.2	5.8 ± 1.0 *	0.9 ± 0.1	8.8 ± 1.1 *
	0.40	8.9 ± 1.4 *	1.2 ± 0.1	5.2 ± 0.9 *	0.9 ± 0.1	8.6 ± 1.5 *
	0.80	8.3 ± 1.6 *	1.3 ± 0.3	4.4 ± 0.5 *	1.1 ± 0.2	8.4 ± 1.1 *
**AaF13**	0.05	13.9 ± 1.8	1.0 ± 0.1	6.0 ± 0.5 *	0.7 ± 0.1	10.8 ± 1.6 *
	0.10	12.2 ± 1.4	1.1 ± 0.0	5.1 ± 0.7 *	0.9 ± 0.1	9.4 ± 2.3 *
	0.20	11.1 ± 1.0 *	1.3 ± 0.3	4.2 ± 0.9 *	1.0 ± 0.1	8.3 ± 1.7 *
	0.40	9.1 ± 1.3 *	1.6 ± 0.3 *	3.3 ± 0.6 *	1.1 ± 0.1	6.4 ± 2.9 *
	0.80	8.7 ± 2.3 *	1.8 ± 0.2 *	2.8 ± 0.3 *	1.4 ± 0.1 *	5.8 ± 1.2 *
**AaF16**	0.05	13.8 ± 1.6	1.3 ± 0.1	5.4 ± 1.4 *	0. 8 ± 0.3	7.5 ± 1.4 *
	0.10	10.4 ± 1.5 *	1.5 ± 0.4 *	3.1 ± 0.3 *	1.2 ± 0.6	6.5 ± 1.2 *
	0.20	7.6 ± 0.7 *	2.4 ± 0.4 *	2.6 ± 0.6 *	1.6 ± 0.2 *	4.9 ± 0.9 *
	0.40	4.2 ± 0.6 *	2.7 ± 0.3 *	1.4 ± 0.2 *	2.0 ± 0.2 *	3.5 ± 0.8 *
	0.80	2.2 ± 0.3 *	3.8 ± 0.7 *	0.8 ± 0.7 *	3.5 ± 0.7 *	2.1 ± 0.7 *

All data are means ± S.E.M. (*n* = 6) and represent the rate increment of each cytokine respect to basal level in ear mice without treatment with Vehicle (No-TPA, no-inflammation). TPA = 12-orto-Tetradecanoylphorbol-13-acetate; DEX = dexametasone; AaAc = Acetonic extract from *A. angustifolia*; AaF13 = Fraction with β-sitosteryl glucoside and stigmasterol; AaF16 = 3-*O*-(6'-*O*-palmitoyl)-β-d-glucopyranosyl sitosterol. * *p* < 0.05 compared with TPA group.

### 2.4. Effect of A. angustifolia on the Cellularity from Ear Edema Induced by TPA

Acute topically application of TPA as was used in this protocol, inducing a skin inflammation characterized by edema (Table 2R, 10×) and infiltration of inflammatory cells (Table 2R, 40×). Cross sections made from punch ears exposed to TPA and different doses of AaAc extract, AaF13 and AaF16 fractions show differences in the thickness of the histological structures (Table 2). Observations done with the 10× objective, revels the inflammatory process (edema) mainly between the skin and cartilage.

**Table 2:** Histopathology analysis of alterations induced by TPA and the protector effect of the different concentrations (0.05, 0.1, 0.2, 0.4 and 0.8 mg/ear) of extracts (AaAc, images **A**–**E**), AaF13 (images **F**–**J**) and AaF16 (images **K**–**O**) fractions from *Agave angustifolia*. BASAL = NO-TPA, DEX = dexamethasone and VEHICLE (ears with TPA).

Concetration (mg/ear)	Treatment	0.05	0.1	0.2	0.4	0.8
**AaAc**		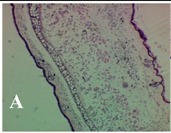	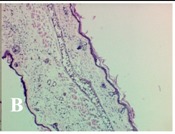	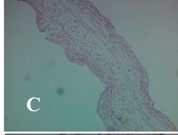	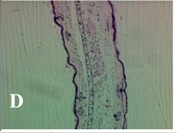	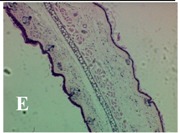
**AaF13**		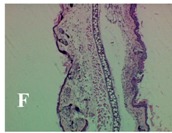	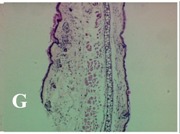	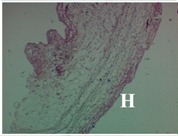	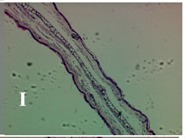	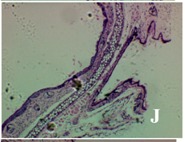
**AaF16**		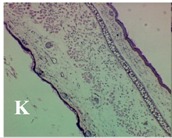	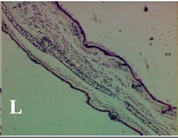	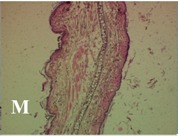	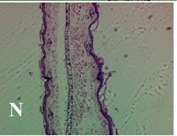	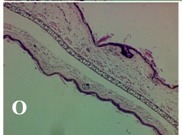
		**BASAL**	**DEX**	**VEHICLE**	**PANEL 10×**	
**Controls**		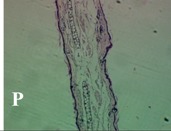	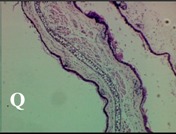	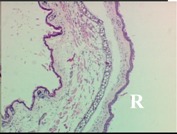		
**AaAc**		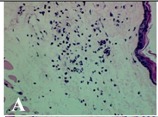	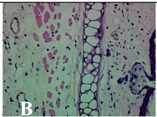	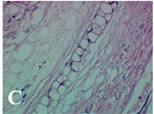	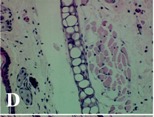	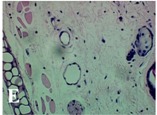
**AaF13**		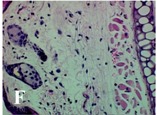	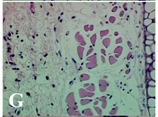	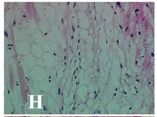	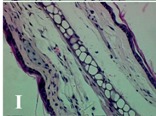	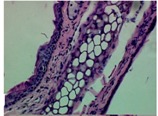
**AaF16**		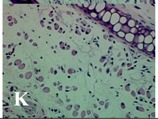	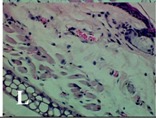	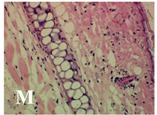	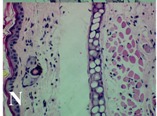	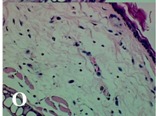
**Controls**		**BASAL**	**DEX**	**VEHICLE**	**PANEL 40×**	
		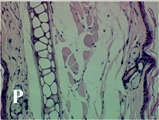	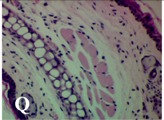	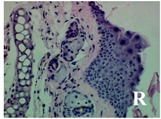		

TPA and low concentration (0.05, 0.1 and 0.2 mg/ear) of the extract and two fractions, show the maximum thickness (Figure 2A–H,K,L,M, 10×) in contrast with those which received concentration of 0.4 and 0.8 mg/ear of AaAc extract (Figure 2D,E, 10×), AaF13 (Figure 2I,J, 10×) and AaF16 (Figure 2N,O, 10×) fractions. In these cases, the tissue return to normal structure comparable with the basal or dexametasona treated mice (Figure 2P,Q, 10×). The observations done with the 40× objective reveled an important infiltration of mononuclear inflammatory cells within the skin and cartilage, mainly in the positive control (vehicle and TPA) (Figure 2R, 40×) and the ears from animals treated with TPA and low concentrations (0.05, 0.1 and 0.2 mg/ear) (Figure 2A–H,K,L,M, 40×). The mice treated with 0.4 and 0.8 mg/ear of the extract and fractions show the lowest level of mononuclear infiltration (Figure 2D,E,I,J,N,O, 40× ) comparable with the basal mice (Figure 2P, 40×) been even more significant than the mice treated with dexametasona (Figure 2Q, 40×).

*Agave angustifolia* is a plant resource of great economic and cultural importance in Mexico [1,2,3]. The widespread medicinal use as anti-inflammatory is justified by the phytochemical and pharmacological antecedents of the family Agavaceae. To date there is only one report in the literature on the pharmacological activity of *Agave angustifolia* demonstrating an anti-inflammatory effect [8]. In contrast, other species like *Agave americana* have been describe extensively in said activity. It was demonstrated that aqueous extract administered orally in doses of 200 and 300 mg/kg had anti-inflammatory effect due to the presence of hecogenin and tigogenin on footpad edema induced by carrageenan [10]. The decoction of *Agave intermixta* leaves, also induced a decrease of footpad edema induced by carrageenan, the treatment consisted of the oral administration of 300 and 500 mg/kg [10]. In the model of mouse ear edema induced with TPA was also observed the anti-inflammatory activity with decreased activity of the enzyme myeloperoxidase [11].

In this paper we showed that the whole extract and fractions, from the “piña” of *Agave angustifolia* are able to decrease acute inflammation of the ear mouse induced with TPA. Despite the mechanism of action by which TPA induces inflammation is not entirely clear, this substance is able to induce the release of eicosanoids, stimulating the enzyme phospholipase A2, which causes the release of arachidonic acid and prostaglandins, mainly PGE2. In the assay of local inflammation induced with TPA, this causes fluid extravasation from the vascular space, with a consequent outflow of proteins and leukocyte infiltration [12,13,14,15,16]. This model is widely used in the evaluation of potential products for anti-inflammatory activity, in which TPA induces skin inflammation with subsequent hyperproliferative response in animals, this simulate in some respects to psoriasis, which underlies an immunophatological frame, that is consistent with the use that is given to *A. angustifolia* in southern Mexico [6,7].

By using this model of acute inflammation, the extract and fraction of *A. angustifolia* caused changes in the concentration of various cytokines, both pro-inflammatory (TNF-α, IL-1β and IL-6) and anti-inflammatory ones (IL4 and IL-10). While the extract and two fractions from A. angustifolia analyzed were able to exert an immunomodulatory effect it was AaF16 fraction which showed the highest activity because it induced significant concentration dependent decrease in the index level of pro-inflammatory cytokines (TNF-α, IL-1β and IL-6) and an increase, also concentration-dependent anti-inflammatory cytokines such as IL-4 and IL-10. In other reports it has been demonstrated that TPA is able to cause a significant increase in the concentration of cytokines TNF-α, IL-1β and IL-6 in biopsies of the mouse ear, although topical administration schedule were sub-chronic, in which the application of TPA was daily for 6 days [17].

The pro-inflammatory cytokines play an important role in the defense mechanisms of the individuals in the inflammatory response in both acute and chronic process. TNFα for example, is crucial for the innate response; however the deregulation of this cytokine mediated signaling, is important in the pathogenesis of various chronic diseases. The IL-6 is an essential mediator of the host response during the acute phase reactions, and IL-1β is produced mainly by macrophages, monocytes and T cells and involves the defense response against infection. The uncontrolled increase in the production of these cytokines has been implicated in several chronic degenerative diseases [18]. Furthermore, IL-10 is part of the homeostatic control mechanism, which modulates the degree and duration of the inflammatory response; it has the ability to selectively block the expression of genes encoding pro-inflammatory cytokines. The IL-10 influences the activity of neutrophils, during the acute phase of inflammation, immune response activation; causes, leading to angiogenesis and restoration of tissues once exceeded the acute phase inflammatory response, among other things [19].

Histological analysis of mouse ear tissue exposed to TPA showed the presence of mononuclear and polymorphonuclear leucocytes, such as monocytes, eosinophils and neutrophils, which are aggregated and bonded to the walls of blood vessels on the range of 4 to 6 h after application of the compound [12]. In this work, samples for histological analysis were collected 6 h after stimulating with TPA, as seen in Figure 2, the TPA was able to induce extravasation of fluids as well as generating edema cell infiltration like polymorphonuclear and mononuclear cells, especially in the space between the skin and cartilage of the ear. The local inflammatory process induced by TPA was counteracted in the animals receiving treatment with Dexamethasone, which is a steroidal anti-inflammatory drug widely used as the validation for the pharmacological model [12]. In regard to treatments with *A. angustifolia*, they were able to decrease the swelling and the leukocyte infiltration consistent with monocytes and polymorphonuclear cells (Figure 2), particularly the highest concentration (0.8 mg/ear) of AaF16 that induced a more effective response compared to the control drug. Therefore it can be assumed that the ear tissue response to harmful agent (TPA) is mediated by a defense mechanism that involves the innate response, such as cellular mediators like cytokines. This damage is controlled by the anti-inflammatory and immunomodulatory effects of *A. angustifolia*. This showed that the active fraction was able to reduce cell damage (edema and leukocyte infiltration) and the concentration of pro-inflammatory cytokines, presumably by stimulating the release of IL-10. The effects described above can be explained by the presence of secondary metabolites synthesized by *A. angustifolia*. For example: steroidal saponin (25*R*-5α-spironstan-12-one-3β-ol-3-*O*-[β-d-xylopyranosyl-(1→4)-β-d-galactopyranosyl-(1→3)-{β-d-xylopyranosyl-(1→3)-β-d-glucopyranosyl-(1→2)}-β-d-glucopyranoside]) obtained from the methanol extract and the saponin fraction of this plant proved to exhibit marked anti-inflammatory, analgesic activities [8].

In this work, the fractionation resulted in the isolation and structural elucidation of other compound which has shown anti-inflammatory activity such as: 3-*O*-[(6'-*O*-palmitoyl)-β-d-glucopyranosyl] sitosterol (**1**). For the present work, the isolation of this compound is important because it is the first time that it is reported for genus *Agave*. The significance of this finding is that it has been reported in the literature, where the mixture of β-sitosterol and 3-*O*-[(6'-*O*-palmitoyl)-β-d-glucopyranosyl] sitosterol is effective in modulating the behavior of T-helper cells [20]. It has even been possible to establish that there is a synergic effect between both components, being critical the presence of 3-*O*-[(6'-*O*-palmitoyl)-β-d-glucopyranosyl] sitosterol for modulating the immune response [21].

We conclude that *A. angustifolia* is a species that has anti-inflammatory compounds in auricular edema model of mouse caused by TPA. Despite being a simple model, you can get a clear and significant pharmacological response to treatment with the extracts and fractions from *A. angustifolia*. This model has been extensively validated by using topically cyclooxygenase inhibitors such as indomethacin, aspirin and piroxicam, although not active when administered orally [11]. A perspective of this work is to use a standardized fraction based on the active compounds described in a useful phytomedicine in the treatment of chronic degenerative diseases, inflammatory background, such as rheumatoid arthritis.

## 3. Experimental Section

### 3.1. Plant Material

*Agave angustifolia* was collected in Yautepec, Morelos (18°49'33.3"N and 99°06'21.98"W; 1120 m above mean sea level), in a controlled culture belonging to the Mexican Company “Yautli” (a distilling company), that are the depositaries of the registers of identity of the biological specimens.

### 3.2. Animals

ICR male albino mice weighing 25–30 g, (Harlan, Mexico D.F., Mexico) were used for this assay. All animas were housed eight per cage and were maintained under laboratory conditions at 25 °C, with a normal 12 h:12 h light/dark schedule (lights on at 7:00 a.m.) and free access to water and food (pellets, Harlan rodent lab diet). The mice were allowed 3 weeks to adapt to the laboratory environment prior to experiments. Experiments were carried out between 8:00 a.m. and 12:00 p.m. All studies were conducted in accordance with official Mexican norm NOM-062-ZOO-1999 (technical specifications for production, care, and use of laboratory animals). This research study was approved by the ethical committee of the Mexican Social Security Institute (R-2010-1701-63). Minimum number of animals and minimum duration of observation required to obtain consistent data were employed.

### 3.3. Extraction and Isolation

The “piña” (stems or central head) of *Agave angustifolia* were chopped, freeze-dried and ground. The dried plant material was macerated in acetone for 48 h (1.1 kg/8.5 L). The acetone extract (AaAc) was concentrated at low temperature and reduced pressure to obtain a yield of 3.2% (35.2 g). This extract (10 g), was chemically separated by using the method of column chromatography (50 × 2.0 cm) with silica-gel 60 (0.2–0.5 mm; Merck, Darmstadt, Germany). The mobile phase consisted of gradient chloroform: acetone: methanol (100:0:0 to 0:50:50), 32 fractions were obtained.

The biological assay indicated that two fractions were the most active, which were labeled as AaF13 (86.1 mg, 0.86% of the acetone extract) and AaF16 (244.9 mg, 2.44% of the acetone extract). Chemical analysis using thin layer chromatography with commercial standards and comparing with AaF13, was identified the presence of two main compounds. However the AaF16 presented a single compound, which was subjected to ^1^H and ^13^C nuclear magnetic resonance (NMR).

### 3.4. 12-Ortho-Tetradecanoylphorbol-13-Acetate (TPA)-Induced Mouse Ear Edema Assay

In order to evaluate the effect produced by *A. angustifolia* on the mouse ear edema assay, six animals per group were used. From each animal, the left ear topically applied all treatments respectably and the right ear of each mouse was used as control, administering 70% ethanol that was also used as vehicle. The application of vehicle and the treatment was of 10 µL in the inner ear and 10 µL in the external ear. The treatments that were employed were: the negative control group that received 2.5 μg/ear of TPA (Sigma-Adrich, St. Louis, MO, USA), the positive control group with dexamethasone (Sigma-Aldrich,) to 0.1 mg/ear in acetone, and *A. angustifolia* extracts: AaAc, AaF13 and AaF16 were dissolved in 70% ethanol and were administered in different concentrations: 0.05, 0.10, 0.20, 0.40 and 0.80 mg/ear. All treatments were applied 15 min previous to TPA application. The edema was determined as a difference of the weight between the inflamed ear with TPA and the ear without inflammation (vehicle). After 6 h, the mice were sacrificed in a camera with ethylic ether; sections of the central portion of each ear (diameter of 6 mm) were dissected and weighed. The activity was evaluated as difference the ear thickness in milligrams, between left and right ear. Then the percentage of inhibition was resolved as:

Inhibition (%) = (Difference of vehicle group − Treatment group/vehicle) × 100

The results obtained were used to construct a dose-effect curve and calculated the E_max_ and ED_50_ for each treatment of *A. angustifolia*.

### 3.5. Quantification of Cytokines from Mouse Ear

All tissue from all ears of mice was transferred to 15 mL tubes, placed on dry ice, and resuspended in phenyl methyl sulfonyl fluoride (PMSF; Sigma-Aldrich) 0.1 mM in PBS (1 mL/200 mg of tissue). Samples were homogenized (Potter-Elvehjem homogenizer) and then centrifuged for 10 min at 14,000 rpm at 4 °C, the supernatants were transferred to 1.5 mL Eppendorf tubes and were ready for analysis. IL1β, IL-4, IL-6, IL10 and TNFα were measured using ELISA BD OptEIA™ (San Jose, CA, USA), according to the manufacturer’s instructions. Concentrations for each cytokine were calculated from calibration curves using individual recombinant proteins as standards, according to the manufacturer’s instructions. Density (OD) readings were performed at 30 and 60 min of incubation at 450 nm.

The data are shown as an index of elevation or decrement from the cytokines levels compared to the mice group without TPA (no-inflammation or basal condition) and are represented in a concentration-effect curve.

### 3.6. Histology

Ear sections were placed *in toto* in 4% phosphate-buffered formaldehyde solution for 1 day, after the preparations were fixed for 48 h in Zamboni fixative solution (saturated picric acid buffered solution of formaldehyde). At the end, the samples were transferred into % EDTA in acidulated water with 5% formic acid for 48 h to decalcify the bones. The tissue was next embedded in paraffin, sectioned (6 μm) and stained with H & E.

### 3.7. Statistical Analysis

The analysis of normal distribution of the data of each group was made with Shapiro-Wilk test. After the data was analyzed with an analysis of variance (ANOVA) followed by Tukey test. A significant difference was established with respect to control group; when the *p* value was lower than 0.05. Statistical analysis was carried out with a SPSS 11.0 program [22].

## 4. Conclusions

In this study the immunomodulatory effect of the extract of *Agave angustifolia* was demonstrated. Furthermore, it was established that the compounds responsible for this activity were 3-*O*-[(6'-*O*-palmitoyl)-β-d-glucopyranosyl] sitosterol, β-sitosteryl glucoside and stigmasterol.
